# Self-Supervised Multiscale Contrastive and Attention-Guided Gradient Projection Network for Pansharpening

**DOI:** 10.3390/s25082560

**Published:** 2025-04-18

**Authors:** Qingping Li, Xiaomin Yang, Bingru Li, Jin Wang

**Affiliations:** College of Electronic Information, Sichuan University, Chengdu 610017, China; lina_lqp@163.com (Q.L.); libingru0126@163.com (B.L.); jin_w6688@163.com (J.W.)

**Keywords:** pansharpening, multiscale contrastive, attention-guided gradient projection network

## Abstract

Pansharpening techniques are crucial in remote sensing image processing, with deep learning emerging as the mainstream solution. In this paper, the pansharpening problem is formulated as two optimization subproblems with a solution proposed based on multiscale contrastive learning combined with attention-guided gradient projection networks. First, an efficient and generalized Spectral–Spatial Universal Module (SSUM) is designed and applied to spectral and spatial enhancement modules (SpeEB and SpaEB). Then, the multiscale high-frequency features of PAN and MS images are extracted using discrete wavelet transform (DWT). These features are combined with contrastive learning and residual connection to progressively balance spectral and spatial information. Finally, high-resolution multispectral images are generated through multiple iterations. Experimental results verify that the proposed method outperforms existing approaches in both visual quality and quantitative evaluation metrics.

## 1. Introduction

Advances in remote sensing satellite technology have made Earth surface observation possible [1]. However, limited by sensor performance, satellites are unable to capture images that contain both rich spectral and spatial information simultaneously. Instead, they can only acquire low-resolution multispectral (MS) images and corresponding high-resolution panchromatic (PAN) images. The importance of high-resolution multispectral images in fields such as change detection [2], classification [3], and target identification [4] has led to the emergence of the pansharpening technique.

Traditional pansharpening methods are mainly divided into strategies based on component substitution (CS) and multiresolution analysis (MRA). CS methods, such as Brovey [5], principal component analysis (PCA) [6], IHS [7], and GSA [8], project the spectral information of the MS image into a new domain by replacing some or all of the spatial information with data from the PAN image, followed by back-projection Although histogram matching is performed before replacement to reduce spectral distortion, it is still difficult to completely avoid spectral aberrations. MRA methods, such as ATWT [9,10], SFIM based on smoothing filters [11], and the MTF-Matched Filtering-Based Generalized Laplace Pyramid (MTF-GLP) [12], extract spatial information from the PAN image through multiscale decomposition, subsequently injecting this information into the upsampled MS image. However, aliasing effects may cause spatial distortion.

Deep learning methods have become mainstream tools due to their powerful feature extraction capabilities and nonlinear mapping performance. Inspired by the Super-Resolution (SR) technique, Masi et al. [13] treated the pansharpening task as a super-resolution problem, using convolutional neural networks (CNNs) to address it. Subsequently, residual networks (RNs) [14,15], generative adversarial networks (GANs) [16,17,18,19,20] and MSDCNN [21], a multiscale deep convolutional network, were proposed.

Variational optimization methods, which lie between traditional CS/MRA methods and deep learning, consider generalized pansharpening as an optimization problem. The P+XS method [22] achieves pansharpening by extracting spatial information from a panchromatic (PAN) image, which is then injected into a multispectral (MS) image. Wu et al. [23] combined variational optimization with deep CNNs to enhance the model’s generalization ability, subsequently proposing a pansharpening framework based on low-rank tensor complementation [24]. In addition, meta-heuristic algorithms [25,26] are also widely used in generalized pansharpening tasks due to their superior performance in large-scale search spaces.

Under the variational optimization framework, it is assumed that a multispectral (MS) image is a reduced-quality version of a high-resolution multispectral (HRMS) image, while a panchromatic (PAN) image is a linear combination of the bands of the HRMS image. Based on this assumption, this paper proposes two optimization problems for HRMS image reconstruction, which constrain the generation of HRMS images using the information from both MS and PAN images, respectively.

Although existing variational optimization methods have shown significant effects on pansharpening, there are still several issues that urgently need to be addressed:

(1) Modal differences between spatial and spectral information lead to inconsistencies in information representation and extraction, resulting in poor fusion performance.
(2) During the optimization process of HRMS images, the high-frequency noise in MS images is not considered in spectral optimization, leading to an increase in artifacts in the reconstructed image.
(3) Balancing spectral and spatial information: Overemphasizing one aspect may lead to a decrease in the overall quality of the final reconstructed image.

To address these challenges, this paper applies contrastive learning to the pansharpening task by introducing an innovative method that combines self-supervised multiscale contrastive learning with attention-guided deep gradient projection (MCAGP).

The method first designs a Spectral–Spatial Universal Module (SSUM) for depth gradient projection networks, combining the depth prior to design spectral enhancement blocks (SpeEBs) and spatial enhancement blocks (SpaEBs). These blocks are applied serially and stacked alternately in the depth gradient projection network to solve the two optimization problems step by step.

Additionally, a multiscale contrastive learning strategy is applied to optimize the spatial information of PAN images. In this strategy, the high-frequency components of PAN images are considered positive samples, while those of MS images are treated as negative samples. This method strengthens the SpaEB’s focus on the spatial features of PAN images while also enhancing the SpeEB’s ability to preserve the spectral properties of MS images.

Finally, a contrastive loss function based on contrastive learning is applied to effectively balance spatial and spectral features by maximizing the similarity between positive samples while minimizing the similarity between negative samples, with model performance further enhanced by incorporating L1 loss.

The experimental results demonstrate that the MCAGP method surpasses both traditional and contemporary advanced methods in terms of visual quality and performance metrics, offering a novel approach to the pansharpening field.

The contributions of this paper are summarized as follows:

(1) Combining contrastive learning with deep gradient projection within a variational optimization framework: this method reduces modal differences by contrasting high-frequency features, strengthens the task focus of the spectral and spatial enhancement blocks, improves feature consistency and reconstruction quality, and overcomes conflicts between modalities through independent optimization strategies.
(2) Introducing a Spectral–Spatial Universal Module (SSUM) combined with depth priors: This module is extended to spectral and spatial enhancement blocks, effectively solving the dual optimization problem. Through channel-space attention guidance and multilevel residual connections, it balances spatial and spectral features.
(3) Designing a multiscale contrastive learning strategy: this strategy introduces contrast loss to filter out noise in MS images, allowing the model to perform well in both full-resolution and reduced-resolution tasks.

The structure of the paper is as follows: Section 2 provides a review of related work; Section 3 describes the MCAGP method in detail; Section 4 presents the experimental results; and Section 5 presents the conclusions.

## 2. Related Work

### 2.1. Self-Supervised Learning

Self-supervised learning (SSL) generates labels using the data themselves to train the model without manual labeling, thus providing a significant advantage in areas where labeling is costly. Its successful applications in natural language processing (NLP) and computer vision (CV), such as image colorization [27,28] and super-resolution [29], demonstrate that SSL is able to efficiently extract structural, contextual, and semantic features from data. In the field of pansharpening, SSL shows great potential. Xing et al. [30] proposed a cross-predictive diffusion model (CrossDiff) to explore self-supervised representations in panchromatic sharpening; Ruben et al. [31] designed a self-supervised double-U network (W-NetPan); and He et al. [32] developed a self-supervised pansharpening method based on spectral super-resolution (sSRPNet). These studies show that SSL offers innovative ideas for panchromatic sharpening tasks, significantly enhancing performance.

### 2.2. Contrastive Learning

Contrastive learning has garnered significant attention, with its core idea being to enhance the mutual information of learned representations by reducing the distance between anchor and positive samples in latent space while pushing negative samples away [33,34,35,36,37,38]. The construction of positive and negative samples is key to contrastive learning. In the field of image super-resolution, positive samples are typically real images, and negative samples are degraded or other images [39,40,41]. SimCLR [34] utilizes data augmentation (e.g., cropping, flipping, and color dithering) to generate pairs of positive samples and learns their similarity through contrast loss. MoCo [35] introduces a momentum encoder and dynamic queue, effectively addressing the problem of balancing positive and negative samples and making representation learning more robust. In the pansharpening field, Zhou et al. [42] enforce contrastive learning to constrain the distance between the restored features and the ground truth, performing distillation to promote the learning of consistent features.

## 3. Proposed Method

**Figure 1:**
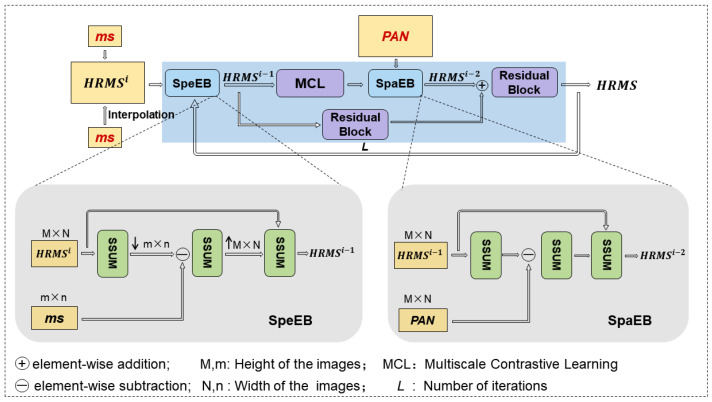
Overall framework diagram of MCAGP.

This subsection describes in detail the proposed pansharpening method MCAGP, whose overall framework is illustrated in Figure 1 and Algorithm 1. In this figure, ms denotes the low-resolution multispectral image, PAN denotes the high-resolution panchromatic image, and HRMS refers to the final high-resolution multispectral image.
**Algorithm 1:** MCAGP Forward Pass
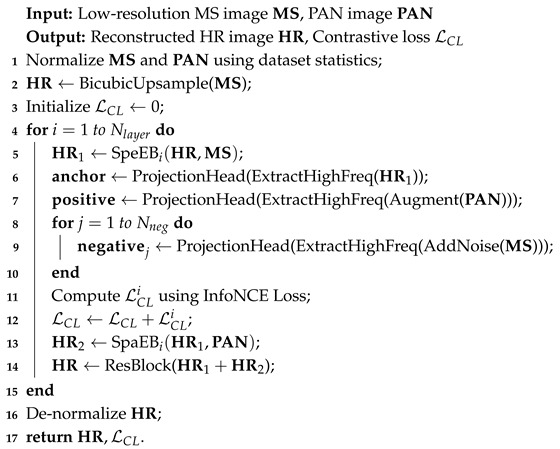


The framework of MCAGP consists of three key components: a spectral enhancement block (SpeEB), a spatial enhancement block (SpaEB), and a Multiscale Contrastive Learning module (MCL), which are closely coupled through iterative residual learning.

Specifically, the process begins with interpolating the low-resolution MS image to the PAN resolution, obtaining the initial ${HRMS}^{0}$. Both the interpolated MS image and the original MS image are then input into the SpeEB, which is designed to enhance the spectral information by learning and compensating the spectral difference between the upsampled image and the original MS image. The output of SpeEB, denoted as ${HRMS}^{l-1}$, is subsequently passed through the MCL module, where the multiscale contrastive loss is calculated by extracting high-frequency details and constructing positive and negative samples based on data augmentation and noise injection, effectively guiding the network to focus on fine-grained spatial–spectral consistency.

Afterwards, the contrastive-enhanced ${HRMS}^{l-1}$ and the PAN image are jointly fed into the SpaEB, which injects spatial details from the PAN image while preserving spectral consistency. A residual block is embedded after SpaEB to further refine the fused result and compensate for residual errors.

This procedure is repeated over L iterations, with residual connections linking the outputs at each stage to progressively refine the reconstructed HRMS. Through the interaction of spectral enhancement, spatial enhancement, and contrastive learning, the network gradually improves the fidelity of the pansharpened image. The detailed workflow is summarized in the pseudo-code provided, and the interconnection between modules is visually illustrated in Figure 1.

### 3.1. Attention-Guided Gradient Projection

**Problem description:** Suppose the LR image is a degraded version of the HR image, while the PAN image is a linear combination of the bands in the HR image. Therefore, the following formula can be obtained:

(1) $$y_{\mathrm{lr}}=DKx_{\mathrm{hr}}$$

(2) $$y_{\mathrm{pan}}=Fx_{\mathrm{hr}}$$

where $D\in R^{mn\times MN}$ denotes the downsampling matrix, K is the low-pass circular convolution matrix, $F\in S^{B\times b}$ is the spectral response function, and $x_{\mathrm{hr}}$ represents the target high-resolution multispectral image. Since the process of reconstructing the HR image is a typical pathological inverse problem, the direct solution often faces instability. Therefore, in order to constrain the reasonableness of the solution, the following optimization problem with a regularization term is proposed:

(3) $$\min_{x_{\mathrm{hr}}}\;{\;L}_{data}\left(x_{\mathrm{hr}}\right)+\gamma R\left(x_{\mathrm{hr}}\right)$$

where $R\left(x_{\mathrm{hr}}\right)$ is the prior term, which is used to control the smoothness or structure of the $x_{\mathrm{hr}}$ image; traditional optimization is typically hand-tailored, while in deep learning, it is represented as an implicit prior. $L_{data}\left(x_{\mathrm{hr}}\right)=∥y_{\mathrm{lr}}-Dx_{\mathrm{hr}}{∥}_{F}^{2}+\;{∥y_{\mathrm{pan}}-Fx_{\mathrm{hr}}∥}_{F}^{2}$ is the data fidelity term, which is used to constrain the consistency between the $x_{\mathrm{hr}}$ image, the $y_{lr}$ image, and the $y_{pan}$ image; γ is the trade-off parameter, which is used to regulate the relative importance between the regularization term and the data fidelity term.

In order to better utilize the deep learning framework, the generalized pansharpening problem is decomposed into two complementary subproblems: spectral optimization and spatial optimization. This decomposition allows for the independent optimization of spectral and spatial information, with the final goal of reconstructing the HR image formulated as follows:

(4) $$\min_{x_{\mathrm{hr}}}\;f\left(y_{\mathrm{lr}},x_{\mathrm{hr}}\right)+\gamma R_{l}\left(x_{\mathrm{hr}}\right)$$

(5) $$\min_{x_{\mathrm{hr}}}\;f\left(y_{\mathrm{pan}},x_{\mathrm{hr}}\right)+\gamma R_{P}\left(x_{\mathrm{hr}}\right)$$

Inspired by generative GAN algorithms, two generative modules were designed: the spectral enhancement block (SpeEB) and the spatial enhancement block (SpaEB). These two modules implicitly model regularization terms through deep learning in order to optimize both the spectral features and spatial details.

**Spectral enhancement block (SpeEB)**: The focus of the spectral enhancement module is to optimize spectra by reconstructing spectral distributions that are consistent with low-resolution (LR) images.The optimization process of SpeEB consists of the following four steps:

(6) $$\hat{{y_{\mathrm{lr}}}^{\left(m\right)}}=DK{x_{\mathrm{hr}}}^{\left(m-1\right)}$$

(7) $${R_{l}}^{\left(m\right)}=y_{\mathrm{lr}}-\hat{{y_{\mathrm{lr}}}^{\left(m\right)}}$$

(8) $${R_{h}}^{\left(m\right)}=\rho {\left(DK\right)}^{T}{R_{l}}^{\left(m\right)}$$

(9) $${x_{\mathrm{hr}}}^{\left(m\right)}{\mathrm{prox}}_{h_{l}}=\left({x_{\mathrm{hr}}}^{\left(m-1\right)}+{R_{h}}^{\left(m\right)}\right)$$

where ρ is the step size, and ${\mathrm{prox}}_{h_{l}}$ is the proximal operator corresponding to the penalty term $h_{l}\left(\cdot \right)$.

**Spatial enhancement block (SpaEB)**: the spatial enhancement block focuses on spatial optimization, which optimizes the details of spatial information by comparing the linear combination of an HR image and a PAN image. Its optimization steps are as follows:

(10) $$\hat{{y_{\mathrm{pan}}}^{\left(m\right)}}=F{x_{\mathrm{hr}}}^{\left(m-1\right)}$$

(11) $${R_{p}}^{\left(m\right)}=y_{\mathrm{pan}}-\hat{{y_{\mathrm{pan}}}^{\left(m\right)}}$$

(12) $${R_{h}}^{\left(m\right)}=\rho {R_{p}}^{\left(m\right)}F^{T}$$

(13) $${x_{\mathrm{hr}}}^{\left(m\right)}{\mathrm{prox}}_{h_{p}}=\left({x_{\mathrm{hr}}}^{\left(m-1\right)}+{R_{h}}^{\left(m\right)}\right)$$

where ρ is the step size, and ${\mathrm{prox}}_{h_{p}}$ is the proximal operator corresponding to the penalty term $h_{p}\left(\cdot \right)$.

**Figure 2:**
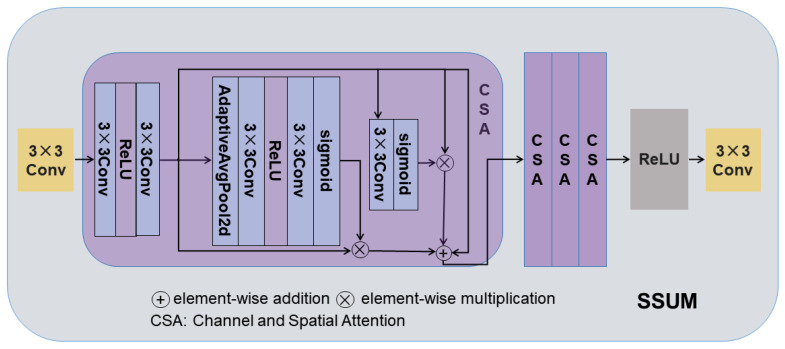
Spectral–Spatial Universal Module (SSUM) framework diagram.

**Spectral–Spatial Universal Module (SSUM)**: The detailed structure of the Spectral–Spatial Universal Module (SSUM) is illustrated in Figure 2. To further enhance the fusion efficiency of spectral and spatial information, this paper introduces the SSUM module between the spectral enhancement block (SpeEB) and the spatial enhancement block (SpaEB), aiming to achieve the unified extraction and enhancement of spectral and spatial features. Specifically, SSUM incorporates both channel attention and spatial attention mechanisms, which effectively guide the network to selectively focus on spectral attributes and spatial details, thereby improving the feature representation capability. In the overall framework, SpeEB mainly leverages the residual information between the low-resolution multispectral (MS) image and the interpolated high-resolution MS image to compensate for the spectral distortion caused by upsampling. Conversely, SpaEB focuses on utilizing the spatial structural details contained in the PAN image and compensates for the spatial resolution loss via a residual back-projection strategy. Although both SpeEB and SpaEB share the same SSUM structure as the basic unit for feature mapping and residual feedback, they achieve functional decoupling and complementarity in terms of input design and residual information utilization. This ensures a well-balanced optimization between spectral fidelity and spatial detail enhancement. Furthermore, the structural versatility and efficiency of SSUM enable feature sharing and collaborative optimization between SpeEB and SpaEB, significantly improving the overall quality of feature representation and computational efficiency.

### 3.2. Multiscale Contrastive Learning

**Figure 3:**
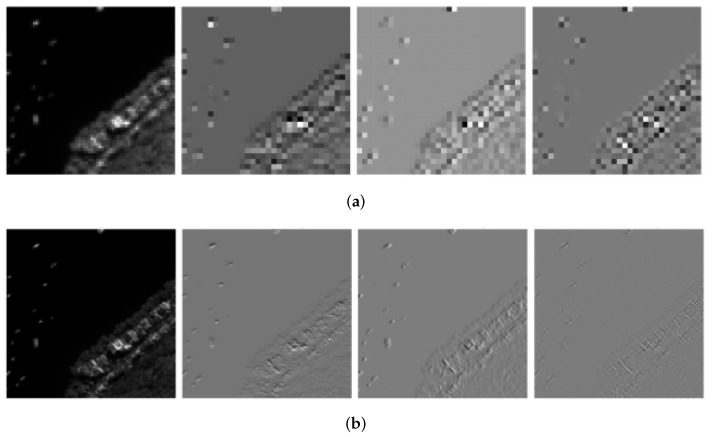
Comparison of the high-frequency portion of an MS image and a PAN image: (**a**) shows the obvious noise in the high-frequency portion of the MS image; (**b**) demonstrates the rich and clear spatial details in the high-frequency portion of the PAN image. (**a**) High-frequency portion of a MS image (from left to right, they are as follows: MS-Original, MS-LH (Vertical High), MS-HL (Horizontal High), and MS-HH (Diagonal High)). (**b**) High-frequency portion of a PAN image (from left to right, they are as follows: PAN-Original, PAN-LH (Vertical High), PAN-HL (Horizontal High), and PAN-HH (Diagonal High)).

In the reconstruction of remote sensing images, MS images have poor spatial quality with significant high-frequency noise (Figure 3a). In contrast, PAN images have clear high-frequency spatial details (Figure 3b). Thus, MS images mainly contribute spectral information, while PAN images provide high-quality spatial details. This division prevents artifacts caused by mixing MS image noise with PAN image details.

**Figure 4:**
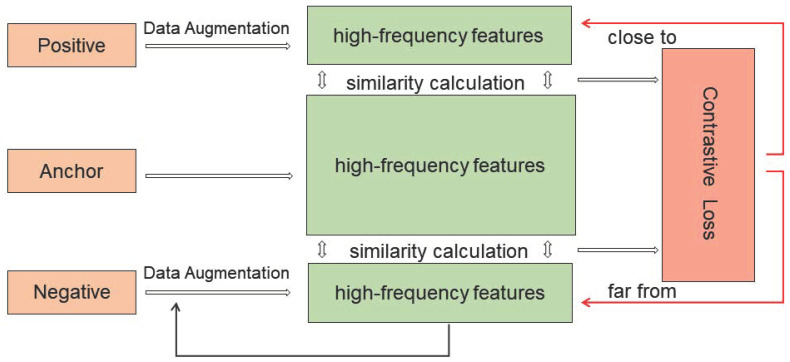
Multiscale contrastive learning framework. Positive samples are generated with multiscale high-frequency components from PAN, anchor samples are pairs of scale high-frequency components after reconstruction with spectral enhancement blocks, and negative samples are multiscale high-frequency components after LRMS interpolation to generate multiple negative samples.

To this end, discrete wavelet transform (DWT) is introduced in this paper to extract the multiscale high-frequency features of PAN and MS images. DWT is able to capture the spatial details in multiscale and multidirectional forms by decomposing the images into low and high-frequency subbands. The multiscale contrastive learning framework is shown in Figure 4. Specifically, the following applies.

**Anchor sample:** The reconstructed image generated via the SpeEB is used to extract its multiscale high-frequency features through DWT, with low-dimensional embedded features generated by global pooling and linear projection.

(14) $$Z_{anchor}=P\left(G\left(\mathrm{DWT}\left(x_{\mathrm{hr}}^{\mathrm{SpeEB}}\right)\right)\right)$$

where G(·) denotes global pooling, G(·) denotes linear projection for mapping high-dimensional features to low-dimensional potential space, and $x_{\mathrm{hr}}^{\mathrm{SpeEB}}$ denotes the HR image generated via the spectral enhancement module.

**Positive sample:** The PAN images are acquired through spatial matching, with multiscale high-frequency features extracted after data enhancement (e.g., random flip and rotation) and the embedded features generated using the same process as for the anchor samples.

(15) $$Z_{pos}=P\left(G\left(\mathrm{DWT}\left(\mathrm{Augment}\left(y_{\mathrm{pan}}\right)\right)\right)\right)$$

Augment(·) represents data enhancement operations such as random flipping and rotation.

**Negative sample:** Extracted from the upsampled MS image, diverse negative samples are generated by adding Gaussian noise, extracting their high-frequency features and mapping them to the low-dimensional space. Through multiple negative samples, the distance between the anchor point and negative samples is enlarged to improve the discriminative ability.

(16) $$Z_{neg}^{i}=P\left(G\left(\mathrm{DWT}\left(\mathrm{AddNoise}\left(y_{\mathrm{lr}}^{↑}\right)\right)\right)\right)$$

where $y_{\mathrm{lr}}^{↑}$ denotes the LR image after upsampling through the interpolation operation, AddNoise(·) denotes the addition of random Gaussian noise to the high-frequency portion of the MS image, and *i* denotes different instances of negative samples.

**Multiscale contrastive learning (MCL):** In the proposed MCAGP framework, a multiscale contrastive learning (MCL) module is introduced. As illustrated in Figure 4, the complete process of positive and negative sample construction, high-frequency feature extraction, and contrastive loss computation is clearly presented, providing readers with a detailed understanding of the implementation and functionality of this module.

The core idea of the MCL module is to guide the network to focus more on the consistency of spatial–spectral details during training by constructing positive and negative sample pairs. Specifically, multiscale high-frequency features are first extracted from the output $HRMS^{l-1}$ of the SpeEB module, which serves as the anchor samples. Subsequently, a data augmentation strategy—including rotation, flipping, color jittering, and other transformations—is applied to the PAN image to generate positive samples. Their multiscale high-frequency features are also extracted. In order to provide effective contrastive information, multiple negative samples are further generated by injecting Gaussian noise into the multispectral image MS, followed by high-frequency feature extraction.

In the feature space, the similarity between the anchor features and the positive features is maximized (i.e., bringing them closer), while the similarity between the anchor features and the negative features is minimized (i.e., pushing them apart). This forms the positive–negative contrastive training objective, where the similarity measurement is implemented using the InfoNCE loss function.

It is noteworthy that the high-frequency feature extraction in the MCL module not only focuses on single-scale texture information but also leverages multiscale spatial details obtained via discrete wavelet transform (DWT). This ensures the effectiveness of contrastive loss across different scales. Additionally, the generation process of positive and negative samples incorporates diverse data augmentation and noise injection strategies, effectively enhancing the model’s discriminative ability and robustness.

**Residual connection and information balance:** To avoid the loss of spectral information due to the over-reliance of the model on the spatial features of the PAN image and to improve the fusion efficiency of spectral and spatial features, this paper introduces a multi-stage residual connection mechanism between the SpeEB, the SpaEB, and the subsequent residual blocks, which progressively accrues the features of each stage and realizes the dynamic balance between the spectral and spatial information.

### 3.3. Loss Functions

**L1 loss:**

(17) $$L_{L1}=∥x_{\mathrm{hr}}^{\mathrm{pred}}-x_{\mathrm{hr}}^{\mathrm{gt}}∥$$

**Contrast loss:** the InfoNCE loss [33,34,35] is used.

(18) $$D=\sum_{i=1}^{K}\exp\left(\frac{\mathrm{sim}(Z_{\mathrm{anchor}},Z_{\mathrm{neg}}^{i})}{\tau }\right)$$

(19) $$L_{\mathrm{InfoNCE}}=-\log\left(\frac{\exp\left(\frac{\mathrm{sim}(Z_{\mathrm{anchor}},Z_{\mathrm{pos}})}{\tau }\right)}{\exp\left(\frac{\mathrm{sim}(Z_{\mathrm{anchor}},Z_{\mathrm{pos}})}{\tau }\right)+D}\right)$$

where $Z_{anchor}$ is the feature representation of the anchor sample, $Z_{pos}$ is the feature representation of the positive sample, $Z_{neg}^{i}$ is the feature representation of the *i* negative sample, with a total of *K* negative samples, τ is a given temperature parameter, which is used to regulate the scaling range of similarity, and $sim\left(a,b\right)$ is the similarity function, which is commonly used to measure the similarity between feature vectors by dot product or cosine similarity.

In the implementation, the dot products of positive and negative samples are batch-processed and spliced by columns to form a logits matrix, where the first position is a positive sample and the rest are negative samples. Cross-entropy loss is a reliable and efficient loss function that is widely utilized in deep networks [43,44,45], and the final contrast loss is calculated by cross-entropy loss.

Thus, the total loss function of the model is as follows:

(20) $$L=L_{L1}+\lambda L_{InfoNCE}$$

where λ for the weight hyperparameters, which are used to balance the proportion of the contribution of the $L_{L1}$ loss and the InfoNCE loss.

## 4. Experiments

### 4.1. Datasets and Metrics

To verify the superiority of the proposed method, we conduct experiments on the Rio dataset (source: WV3), Guangzhou dataset (source: GF2), and Indianapolis dataset (source: QB), which all have a scale factor of 4, and the test sets contain the reduced-resolution test set and the full-resolution test set, respectively. As shown in Table 1, The data can be found at GitHub-liangjiandeng/PanCollection.

For the reduced-resolution experiments, we used four commonly used metrics: peak signal-to-noise ratio (PSNR), structural similarity index (SSIM) [46], spectral angle mapping (SAM) [47], and relative unquantized global synthesis error (ERGAS) [48]. For the full-resolution experiments, we use the spectral distortion index (Dλ), the spatial distortion index (D*s*), and the quality of the reference-free mixing (QNR) to assess the quality of the results.

Our MCAGP is implemented in the PyTorch-3.8 framework with Adam optimizer, a learning rate of $5\times 10^{-5}$, L2 regularization, a weight decay factor of $1\times 10^{-4}$, a batch size of 4, and a network depth and width of 64 and 8, respectively. The experiments were performed on MATLAB 2019b and NVIDIA RTX 4050 GPU computers (NVIDIA, Santa Clara, CA, USA). For other deep learning pansharpening methods, we trained the network using the default settings from the relevant papers or code repositories, using the same equipment and PyTorch environment.

### 4.2. Comparison with SOTA Methods

In this section, we compare what we propose in this paper with several state-of-the-art methods, including three traditional methods, i.e., EXP [49], C-GSA [50], and BDSD-PC [51], TV [52], PWMPF [53], and nine deep learning-based methods, i.e., DaViT [54] and its variants, such as paDaViT and rDaViT, LeWin [55], MSDCNN [21], PanFormer [56], SSIN [57], PANNET [58], and PNN [13],. We conducted reduced-resolution and full-resolution experiments on three datasets, with the reduced-resolution following the Wald protocol.

**Figure 5:**
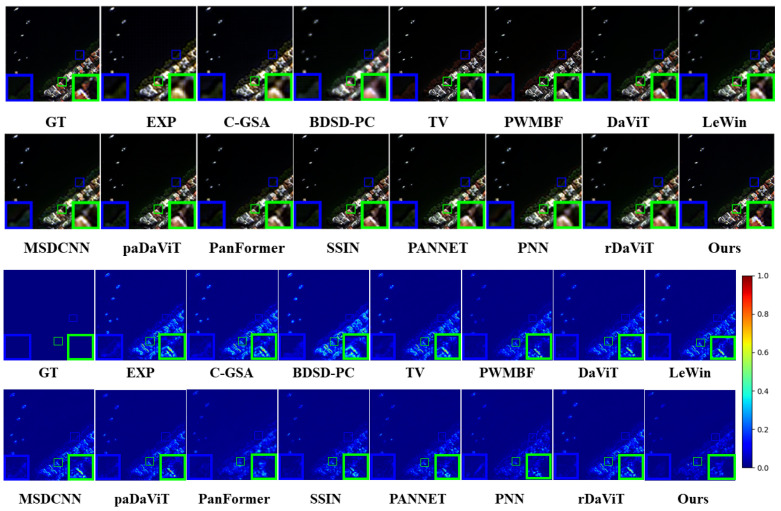
Visualization on the WV3 dataset.

**Results on WV3 dataset**: Table 2 shows the results of quantitative experiments on the WV3 dataset, while Figure 5 provides a visualization of the fused images. Overall, the deep learning-based approach shows significant advantages over the traditional approach. In the resolution reduction experiments, our method is 1.331 dB ahead of the suboptimal method in the PSNR metric, with 0.013 higher in the SSIM metric, indicating a significant improvement in image restoration quality. The restored images are clearer and more natural, with better preservation of details and structures. Our method reduces the spatial angle metric (SAM) by 0.01 and the ERGAS value by 0.458 compared to the suboptimal method, which further indicates that our method achieves a superior balance between preserving spatial details and spectral accuracy. These metrics show that our method effectively recovers the high-frequency details of the image during image reconstruction while reducing the recovery error and enhancing the realism of the image. In the full-resolution experiments, although our Dλ (spectral distortion) is slightly higher than that of other methods, indicating a slight trade-off in spectral recovery, we succeeded in minimizing spatial distortion by optimizing D*s* (spatial distortion). This optimization allowed us to achieve optimal performance in the recovery of spatial details, ensuring high-resolution image recovery. In terms of the final QNR (quality-to-noise ratio) value, our method achieves the best performance, indicating that we have achieved an ideal balance between image quality and noise control and thus ensured the detail and visual quality of the image. In terms of visual effect, our method significantly improves the clarity and detail performance of the image, especially in the detailed presentation of buildings and vegetation, with a sharper restoration effect.

**Figure 6:**
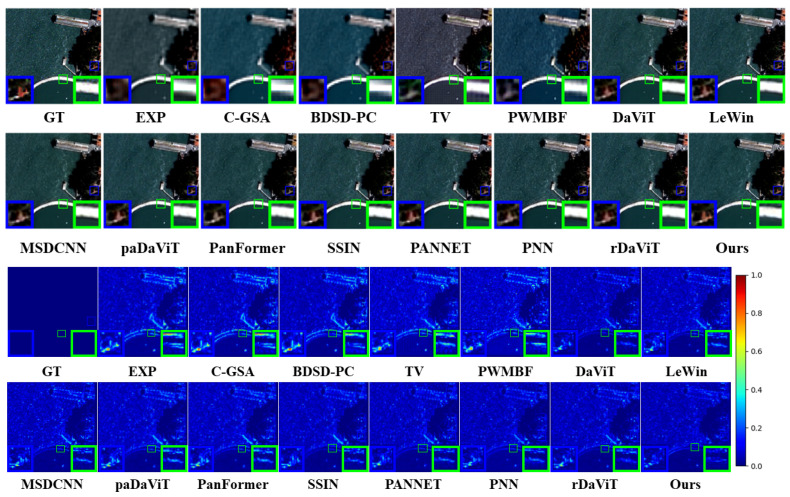
Visualization of the QB dataset.

**Results on the QB dataset**: Table 3 lists the quantitative results on the QB dataset, while Figure 6 demonstrates the corresponding visual effects. Overall, the deep learning-based method outperforms the traditional approach. In the resolution reduction experiments, our method surpasses the suboptimal method by 0.1635 dB in PSNR and 0.022 in SSIM, indicating superior performance in image noise suppression, detail retention, and structure restoration. SAM and ERGAS values are lower than the suboptimal method by 0.012 and 1.095, respectively, suggesting that our method maximizes spectral restoration, preserving the spectral features of the original image and effectively reducing reconstruction errors. In the full-resolution experiments, our method slightly sacrifices spectral distortion (Dλ), but this does not affect overall performance. Our spatial distortion (D*s*) is the lowest among all methods, demonstrating that we minimize spatial distortion during image restoration, ensuring accurate recovery of spatial structure and details. Notably, in the comprehensive QNR (quality-to-noise ratio) metric, our method achieves the best performance, indicating an ideal balance between image quality and noise control.

**Figure 7:**
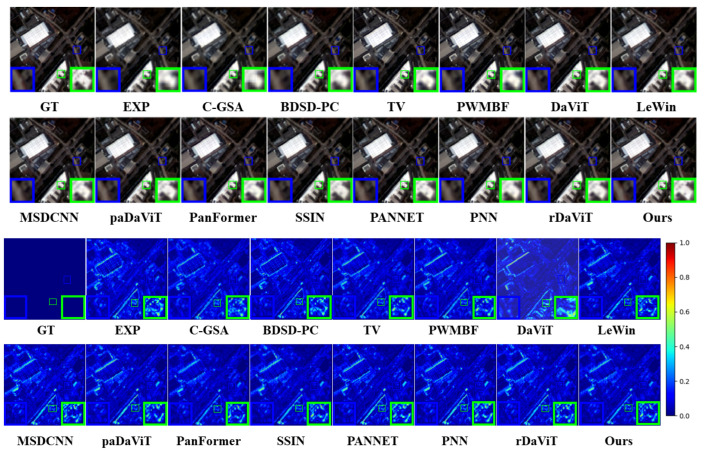
Visualization of the GF2 dataset.

**Results on the GF2 dataset**: Table 4 summarizes the experimental results on the GF2 dataset, while Figure 7 presents a visual representation of the fused images. In the down-resolution experiments, our method outperforms the next best method by 0.075 dB in PSNR and 0.019 in SSIM, demonstrating its superiority in image restoration quality, particularly in detail and contrast preservation. SAM is the lowest among all methods, indicating better spatial restoration performance, and ERGAS is 1.238, which is 0.022 higher than the optimal paDaViT method but still shows better results. In the full-resolution experiments, our method continues to significantly outperform traditional methods, although it performs slightly lower than individual deep learning methods in some metrics, especially in spectral recovery. Overall, our method achieves a balance between spectral and spatial details in image restoration, with superior overall performance. Visually, the fused images exhibit lower noise, fewer artifacts, and sharper details with better contrast.

The performance on the GF2 dataset is not as good as that on the QB and WV3 datasets, mainly due to the noise level in the data, scene complexity, and the stringent demands of the unsupervised full-resolution evaluation protocol on the model’s generalization ability. The GF2 dataset contains more fragmented structures, a mix of vegetation and urban textures, and more pronounced edge aliasing effects, which increase the difficulty of image restoration. Additionally, the performance on the GF2 dataset in the full-resolution experiments is not as good as that on other datasets, partly because the Wald protocol we used has limited applicability to the GF2 dataset. While the Wald protocol works effectively for high-quality commercial sensors such as QB and WV3, it may not hold for GF2, as significant details and noise patterns are lost during the downsampling process, and the generated pseudo-GT exhibits substantial statistical deviation from the true full-resolution images in both spectral and texture domains. Although our method outperforms others in down-resolution experiments, the performance on the GF2 dataset in full-resolution evaluation is slightly worse than on other datasets due to these factors.

### 4.3. Ablation Experiments

To evaluate the contribution of each module in the proposed method, we conducted ablation experiments on the QB dataset by replacing or removing different modules, comparing the experimental results with the final model (Ours) and analyzing the impact of each module on the model performance. The experimental results are shown in Table 5 and analyzed in detail below:

(1) Replacing the SSUM module with regular convolution while removing the contrastive learning part.

In the experimental setup (1), the SSUM module is replaced with regular convolution, with the contrastive learning part removed. Compared with our final model (our approach), PSNR decreased by 8.54%, SSIM decreased by 3.99%, SAM increased by 22.09%, ERGAS increased by 39.50%, and QNR decreased by 0.66%. The results show that regular convolution cannot replace the efficient SSUM module, with the removal of contrastive learning significantly reducing the model’s performance in both down-resolution and full-resolution experiments.

(2) Replacing the SSUM module with regular convolution while retaining only the contrastive learning component.

In experimental setup (2), contrastive learning and its loss function are retained, but the SSUM module is replaced with ordinary convolution. Compared with our approach, PSNR decreased by 8.97%, SSIM decreased by 3.57%, SAM increased by 19.77%, ERGAS increased by 41.66%, and QNR decreased by 0.66%. The results demonstrate the key role of the SSUM module in the model, which can significantly improve the reconstruction quality of image details and effectively reduce errors.

(3) Retaining the SSUM module while deleting the contrastive learning part.

In experimental setup (3), only the SSUM module is used, and the contrastive learning part is removed. Compared with our approach, PSNR decreased by 3.78%, SSIM decreased by 1.05%, SAM increased by 8.14%, and ERGAS increased by 13.95%. Although the SSUM module improves the reconstruction quality, the removal of contrastive learning degrades the model’s performance in the high-resolution reconstruction task; the spectral and spatial properties especially cannot be fully optimized, further validating the importance of contrastive learning.

### 4.4. Discussion of the Loss Function Parameter, λ

To address the different optimization objectives of the two loss functions, we investigated the impact of introducing contrast loss at different stages on model performance, proposing a new strategy that adds contrast loss at a later stage to fine-tune the already established model. In our experiments, we compared two training strategies: one introduced the contrast loss in the whole process (i.e., the method in this paper, with λ = 1); the other trained the model using the L1 reconstruction loss initially to establish the basic image reconstruction capability, followed by gradually increasing the weight of contrast loss until it matched the L1 loss. The training results are shown in Table 6, Table 7 and Table 8.

On the WV3 dataset, our method prioritizes spectral retention, reflected by a lower SAM and Dλ, but with a slight sacrifice in spatial consistency (indicated by the increase in D*s*). In contrast, the two-stage training strategy balances spectral and spatial properties better, though at the cost of a slight reduction in PSNR. For tasks requiring high spectral fidelity, such as surface classification and hyperspectral analysis, our method is more suitable. For higher overall performance, the two-stage strategy can be considered. On the QB dataset, our method offers a better balance between spectral and spatial performance, achieving a superior overall performance index. On the GF2 dataset, the two-stage method strikes a better balance between spatial details and spectral consistency, effectively reducing global error (ERGAS); in full-resolution tests, our method shows better spatial detail recovery and noise suppression.

## 5. Conclusions

In this paper, we have proposed a generative network for depth gradient projection based on self-supervised multiscale contrastive learning and attention guidance that improves the balance of spectral and spatial information. We first proposed an efficient SSUM module based on channel and spatial attention, which was combined with a depth prior and generalized to the depth gradient projection network to form a spectral enhancement block and a spatial enhancement block, which is the basis of our network. Secondly, based on the two proposed optimization problems, we used contrastive learning by using the multiscale high-frequency component of PAN as the positive sample and the upsampled multiscale high-frequency information of MS as the negative samples. This enables the spectral enhancement block and the spatial enhancement block to focus more on their respective optimization tasks; in the end, contrastive loss was applied throughout the process to refine the model, leading to improved reconstruction quality. The experiments demonstrate the superiority of the proposed method in this paper. In the future, research on contrastive learning loss will continue to be strengthened, and it is believed that contrastive learning will have more space for development in the field of pansharpening.

## Figures and Tables

**Table 1:** Dataset Information. B is the number of channels in the multispectral image.

Data	B	MS-Resolution	PAN-Resolution
WV3	8	64	256
QB	4	64	256
GF2	4	64	256

**Table 2:** Test results for the WV3 dataset at reduced and full resolution. (Bold: best; underline: second best).

Method	PSNR ↑	SSIM ↑	SAM ↓	ERGAS ↓	Dλ↓	Ds ↓	QNR ↑
EXP	27.409	0.678	0.135	8.441	0.056	0.156	0.796
C-GSA	31.245	0.853	0.138	5.567	0.102	0.075	0.831
BDSD-PC	31.521	0.873	0.130	5.313	0.063	0.073	0.870
TV	32.381	0.905	0.094	4.855	0.078	0.102	0.829
PWMBF	32.130	0.919	0.097	3.932	**0.028**	0.078	0.894
DaViT	30.950	0.892	0.097	4.465	0.031	0.085	0.887
LeWin	30.591	0.882	0.096	4.694	0.029	0.082	0.892
MSDCNN	30.441	0.884	0.098	4.758	0.035	0.089	0.880
paDaViT	32.392	0.924	0.087	3.761	0.030	0.072	0.901
PanFormer	32.182	0.924	0.085	3.880	0.036	0.083	0.889
SSIN	34.252	0.955	0.070	3.036	0.029	0.075	0.898
PANNET	31.257	0.899	0.091	4.413	**0.028**	0.077	0.898
PNN	29.412	0.857	0.105	5.288	0.035	0.093	0.876
rDaViT	31.213	0.900	0.092	4.337	0.031	0.085	0.888
Ours	**35.583**	**0.968**	**0.060**	**2.578**	0.033	**0.062**	**0.907**

**Table 3:** Test results for the QB dataset at reduced and full resolution. (Bold: best; underline: second best).

Method	PSNR ↑	SSIM ↑	SAM ↓	ERGAS ↓	Dλ↓	Ds ↓	QNR ↑
EXP	28.038	0.682	0.145	11.927	0.079	0.186	0.750
C-GSA	32.057	0.861	0.125	7.530	0.080	0.137	0.794
BDSD-PC	31.920	0.855	0.136	7.648	**0.029**	0.069	0.904
TV	32.174	0.865	0.142	7.690	0.046	0.074	0.884
PWMBF	34.223	0.904	0.118	5.504	0.058	0.068	0.878
DaViT	32.880	0.909	0.114	6.313	0.038	0.115	0.852
LeWin	32.722	0.901	0.119	6.427	0.047	0.102	0.857
MSDCNN	33.060	0.917	0.113	6.183	0.039	0.110	0.855
paDaViT	33.404	0.921	0.108	5.951	0.045	0.098	0.862
PanFormer	33.552	0.914	0.102	5.857	0.048	0.114	0.843
SSIN	33.861	0.930	0.098	5.667	0.044	0.092	0.869
PANNET	33.496	0.920	0.108	5.888	0.053	0.073	0.878
PNN	32.506	0.899	0.121	6.601	0.035	0.106	0.864
rDaViT	33.125	0.912	0.111	6.145	0.039	0.110	0.856
Ours	**35.858**	**0.952**	**0.086**	**4.572**	0.052	**0.045**	**0.906**

**Table 4:** Test results for the GF2 dataset at reduced and full resolution. (Bold: best; underline: second best).

Method	PSNR ↑	SSIM ↑	SAM ↓	ERGAS↓	Dλ↓	Ds ↓	QNR ↑
EXP	31.094	0.794	0.035	2.645	**0.019**	0.167	0.816
C-GSA	33.944	0.895	0.033	1.924	0.053	0.134	0.820
BDSD-PC	33.882	0.894	0.032	1.911	0.049	0.139	0.819
TV	33.900	0.904	0.030	1.598	0.067	0.074	0.865
PWMBF	34.510	0.896	0.031	1.673	0.024	0.076	0.874
DaViT	36.897	0.933	0.022	1.269	0.036	0.054	0.913
LeWin	36.327	0.920	0.024	1.357	0.035	0.049	0.918
MSDCNN	36.166	0.923	0.024	1.368	0.033	0.045	0.923
paDaViT	37.232	0.934	**0.021**	**1.216**	0.038	0.056	0.908
PanFormer	36.483	0.929	0.024	1.315	0.034	0.049	0.919
SSIN	36.411	0.929	0.023	1.330	0.037	**0.041**	**0.924**
PANNET	36.478	0.926	0.022	1.318	0.038	0.043	0.921
PNN	35.616	0.914	0.028	1.461	0.034	0.049	0.917
rDaViT	37.023	0.935	**0.021**	1.247	0.034	0.056	0.912
Ours	**37.307**	**0.954**	**0.021**	1.238	0.046	0.064	0.892

**Table 5:** Results of ablation experiments on the QB dataset. (Bold: best; underline: second best).

	SSUM	CL	PSNR ↑	SSIM ↑	SAM ↓	ERGAS ↓	Dλ↓	Ds ↓	QNR ↑
$\left(1\right)$	×	×	32.795	0.914	0.105	6.380	0.043	0.060	0.900
$\left(2\right)$	×	✓	32.641	0.918	0.103	6.477	**0.039**	0.064	0.900
$\left(3\right)$	✓	×	34.504	0.942	0.093	5.210	0.045	0.080	0.879
Ours	✓	✓	**35.858**	**0.952**	**0.086**	**4.572**	0.052	**0.045**	**0.906**

**Table 6:** Test results of the WV3 dataset introducing contrast loss at different time periods. (Bold: best).

	SSUM	CL	Two-Stage	PSNR ↑	SSIM ↑	SAM ↓	ERGAS ↓	Dλ↓	Ds ↓	QNR ↑
	✓	✓	✓	34.952	0.966	0.062	2.752	0.036	**0.052**	**0.914**
Ours	✓	✓	×	**35.583**	**0.968**	**0.060**	**2.578**	**0.033**	0.062	0.907

**Table 7:** Test results of the QB dataset introducing contrast loss at different time periods. (Bold: best).

	SSUM	CL	Two-Stage	PSNR ↑	SSIM ↑	SAM ↓	ERGAS ↓	Dλ↓	Ds ↓	QNR ↑
	✓	✓	✓	35.661	**0.952**	0.088	4.650	0.059	0.079	0.867
Ours	✓	✓	×	**35.858**	**0.952**	**0.086**	**4.571**	**0.052**	**0.045**	**0.906**

**Table 8:** Test results of the GF2 dataset introducing contrast loss at different time periods. (Bold: best).

	SSUM	CL	Two-Stage	PSNR ↑	SSIM ↑	SAM ↓	ERGAS ↓	Dλ↓	Ds ↓	QNR ↑
	✓	✓	✓	**37.394**	**0.954**	**0.020**	**1.203**	**0.045**	0.071	0.887
Ours	✓	✓	×	37.307	**0.954**	0.021	1.238	0.046	**0.064**	**0.892**

## Data Availability

Data are contained within the article.
